# A prospective study of chemotherapy-induced febrile neutropenia in the South West London Cancer Network. Interpretation of study results in light of NCAG/NCEPOD findings

**DOI:** 10.1038/sj.bjc.6606059

**Published:** 2010-12-21

**Authors:** M Okera, S Chan, U Dernede, J Larkin, S Popat, D Gilbert, L Jones, N Osuji, H Sykes, C Oakley, L Pickering, F Lofts, S Chowdhury

**Affiliations:** 1 Department of Medical Oncology, Guy's & St Thomas' NHS Foundation Trust, Great Maze Pond, London SE1 9RT, UK; 2 Department of Oncology, St George's Hospital NHS Trust, London, UK; 3 The Royal Marsden NHS Foundation Trust, London, UK; 4 Department of Oncology, Epsom and St Helier University Hospitals, London, UK; 5 Department of Oncology, Mayday University Hospital, London, UK; 6 Kingston NHS Trust, London, UK

**Keywords:** neutropenic sepsis, chemotherapy, infection, febrile neutropenia

## Abstract

**Background:**

Chemotherapy-induced febrile neutropenia is a medical emergency complicating the treatment of many cancer patients. It is associated with considerable morbidity and mortality, as well as impacting on healthcare resources.

**Methods:**

A prospective study of all cases of chemotherapy-induced febrile neutropenia in the South West London Cancer Network was conducted over a 4-month period. Factors including demographics, treatment history, management of febrile neutropenia and outcome were recorded.

**Results and conclusi:**

Our results reflect those of the recent National Chemotherapy Advisory Group (NCEPOD, 2008)/National Confidential Enquiry into Patient Outcomes and Death reports (NCAG, 2009) and highlight the need for network-wide clinical care pathways to improve outcomes in this area.

Chemotherapy-induced febrile neutropenia is associated with substantial morbidity, mortality, and healthcare costs (Lyman *et al*, 1998; Crawford *et al*, 2004; Kuderer *et al*, 2006), and is a medical emergency prompting immediate hospitalisation in most cases for assessment and treatment. Management of the underlying cancer may be compromised, as delays and/or dose reductions of subsequent courses of chemotherapy can negatively affect long-term outcomes (Pettengell *et al*, 1992; Lyman, 2005; Chirivella *et al*, 2006; Clamp *et al*, 2008). The risk of febrile neutropenia and its complications published in clinical trials may be underestimated (Dale *et al*, 2003) and not reflect everyday clinical practice. Reasons for this include selection bias and inconsistent reporting of toxicity data. It is also important to recognise that trial data may not reflect the widespread implications, costs and resource strain involved in the management of neutropenic sepsis.

The costs of emergency care, inpatient and intensive care provision, as well as increased demands on nursing and physician time, are all consequences of neutropenic sepsis. Prevention is not always possible, but can be achieved by primary prophylactic use of antibiotics and haematopoietic growth factors (e.g., G-CSF), which have been shown to decrease the incidence and mortality of febrile neutropenia. In a systematic review of 15 randomised controlled trials, use of prophylactic G-CSF led to a 46% decrease in the occurrence of febrile neutropenia (Kuderer *et al*, 2007), and a meta-analysis of randomised controlled trials showed that prophylactic antibiotics resulted in fewer febrile episodes and bacterial infections with a 34% (95% CI: 25–41%) reduction in death (Gafter-Gvili *et al*, 2005).

Underpinning the issues which face the management of febrile neutropenia, is the fact that chemotherapy services must be provided in a safe environment that simultaneously strives for quality. This was highlighted in the 2008 National Confidential Enquiry into Patient Outcomes and Death (NCEPOD) report, which provided a critical review of the care of cancer patients who died within 30 days of receiving chemotherapy in the United Kingdom (NCEPOD, 2008). In all, 83 admissions because of neutropenic sepsis were recorded and significant deficiencies in the level of care identified. Three cases were considered to have received suboptimal care and their deaths directly attributable to chemotherapy-related complications, with delay in treatment of the toxicity contributing to two of the three deaths. Problem areas identified were related to organisational, clinical and patient aspects, and are detailed in Table 1. Delays in admission, prescription and administration of antibiotics, lack of policies and inadequate seniority of medical staff, were deemed to be fundamental areas of weakness requiring urgent improvement.

The National Chemotherapy Advisory Group (NCAG) is a body established in the United Kingdom whose role is to advise the National Cancer Director and Department of Health on the development and delivery of high quality chemotherapy services. Following the concerns raised by NCEPOD, NCAG published a set of recommendations (NCAG, 2009), encouraging service providers to reflect on current practice and implement a step-wise progression towards safer and better cancer care. Specific recommendations relating to management of chemotherapy-related complications were:

- Improved patient information about what they should do in the event of developing a complication
- Provision of an urgent assessment facility with appropriately trained staff
- Network coordination to ensure standardised policies and pathways are in place and are accessible, and to develop close links with A and E departments in neighbouring hospitals
- A 24-h telephone advice from an oncology consultant
- ‘Treat and transfer’ arrangements to be in place if facilities do not have appropriate expertise for inpatient management
- ‘Door to delivery’ time of antibiotic administration for neutropenic sepsis to be within 1 h
- Patients’ treating oncology team to be notified of complications within 24 h
- NICE to develop a national clinical guideline on management and prevention of neutropenic sepsis

To determine our current practice and outcomes and as an aid towards mapping health resource implications, we conducted a prospective study of all admissions with chemotherapy-induced febrile neutropenia in hospitals within the South West London Cancer Network (SWLCN). This is a single cancer network in South East England covering a population of ∼1.4 million people. We analysed our results with particular attention to the findings of the NCEPOD data and recommendations from the NCAG report.

## Materials and methods

Prospective patient data was captured at the seven hospitals of the South West London Cancer Network (Epsom and St Helier University Hospitals, Kingston Hospital, Mayday Hospital, The Royal Marsden Hospital – Fulham Road and Sutton sites and St George's Hospital). All participating hospitals had pre-existing policies for the assessment and management of neutropenic sepsis; however these did not consistently ensure that all patients were managed according to agreed standards, and the policies were not standardised across the network. Over a period of 4 months between May and August 2007, all new hospital admissions of adult patients (aged ⩾18) undergoing systemic therapy for a diagnosis of malignancy and presenting with febrile neutropenia, were documented on a shared database. Cases were identified and screened for inclusion on a daily basis by a nominated physician at each hospital site. Owing to concerns about capturing all patients admitted within the network, there was agreement with the haematology department at each hospital to screen all patients with neutropenia and cross-reference them with admission. Febrile neutropenia was defined as a measured body temperature of ≥38°C and neutrophils <1 × 10^9 ^l^−1^ at first assessment.

Data for demographics, clinicopathological details, previous and current systemic therapy, inpatient management including use of haematopoietic growth factors and clinical outcome was recorded. Data accrual was performed using a unified data collection tool, which consisted of a form with pre-specified fields. Data field groupings included: (1) patient demographics, (2) clinical details, (3) treatment history and (4) outcome data. In total, 58 items were obtained for each case. On completion of the study period, all prospectively completed forms were centrally collated and data transcribed to a centrally held, electronically secure database. The audit committees of each of the participating hospitals approved the study.

## Results

### Patient details

In all, 71 admissions for febrile neutropenia were reported involving 64 patients. In all, 7 patients were admitted on two separate occasions, whereas the remaining 57 were admitted on a single occasion. In all, 38 patients (59%) were female and 26 (41%) were male. The median age was 60 years (range: 20–78 years). In all, 50 (70%) cases were assessed and admitted to one of the two ‘cancer centres’ (St George's Hospital and The Royal Marsden Hospitals) within the cancer network.

### Clinical details

The sites of underlying malignancies are summarised in Table 2. The most common tumour types were breast cancer (18%) and lymphoma (18%). Only 11 (15%) patients admitted with febrile neutropenia were asymptomatic. All other patients had one or more symptoms as detailed in Table 3. Most commonly, patients complained of respiratory (49%) and gastrointestinal (46%) symptoms. Notably, six (8%) patients presented with severe haemodynamic compromise with a recorded systolic blood pressure of <80 mm Hg. The mean presenting neutrophil count at admission was 0.3 × 10^9 ^l^−1^. Information on HDU/ICU admission was not available at the time of data analysis.

### Treatment history

Systemic anti-cancer therapy had been given with palliative intent in 37 (54%) patients, with curative intent (as primary single-modality treatment) in 17 (24%), as adjuvant (post-operative) treatment in 9 (13%) and as neoadjuvant (pre-operative) therapy in 6 (9%) patients. All patients had received conventional cytotoxic chemotherapy. There were no cases due to the sole use of ‘targeted’ treatments, such as monoclonal antibodies or tyrosine kinase inhibitors. The commonest chemotherapy regimens recorded were cyclophosphamide, vincristine, doxorubicin and prednisolone: eight cases (11%), docetaxel: six cases (8%), doxorubicin and cyclophosphamide: five cases (7%), infusional 5-fluorouracil with irinotecan: four cases (6%), and 5-fluorouracil, epirubicin, cyclophosphamide: 3 cases (4%). In all, 44% patients had received anthracyclines as part of their chemotherapy. Febrile neutropenia occurred at a median of 10 days (range: 5–21) after starting the last course of chemotherapy and 36 (50%) episodes occurred after cycles 1 or 2. In all, 11 (15%) patients had an indwelling central venous catheter present. In all, 19 (25%) patients had received antibiotics as primary prophylaxis and 17 (23%) patients had received primary prophylactic G-CSF. Amongst the group that received prophylactic G-CSF, the most common diagnoses were lymphoma (six patients) and sarcoma (four patients).

Almost all (98%) patients in our data series had (level I/II evidence for patient related) risk factors for the development of febrile neutropenia, as described by the American Society of Clinical Oncology (ASCO) and European Organisation of Research and Treatment of Cancer (EORTC) guidelines (Table 4). In all, 39 (54%) patients had advanced stage disease, 25 (35%) patients were ⩾65 years old and 18 (25%) had had a previous neutropenic event. In all, 23 (32%) patients had received systemic therapy with at least 20% risk of febrile neutropenia.

### Outcome data

A total of 45 (63%) patients were admitted directly to a specialist oncology or haematology ward. In all, 21 (30%) were seen in the accident and emergency department first. The median time from arrival to nursing assessment and recording of observations was 10 min (range: 0–135). The median time to first assessment by a clinician was 40 min (range: 0–230). Data on the seniority of admitting clinician was not specifically recorded, however, based on medical rotas within the SWLCN, was likely to be FY1/2 (post-completion medical degree year 1–2) and ST1/2 (post-completion medical degree year 2–4) doctors. The median time from arrival to administration of an antibiotic was 135 min (range: 15–550) 9 out of 50 patients received antibiotics within 60 min (‘time of antibiotics’ data only available for 50 patients). Regarding clinical investigations: chest x-ray was obtained in 62 (87%) patients, urinalysis by dip-stick testing in 55 (71%) cases; blood cultures were obtained in 66 (92%), urine cultures in 52 (73%) and stool cultures in 27 cases (38%).

All patients received intravenous antibiotics. At the time of data analysis, information on whether first-line antibiotics were appropriate and compliant with local policies was not available. In all, 22 (30%) patients required ‘second-line’ treatment, whereby antibiotics were changed after 48 h of persisting intermittent fever of 38^o^C or above. In all, 12 (17%) patients received additional antifungal regimens. In all, 45 (63%) patients received treatment with G-CSF. The median duration of hospital stay was 5 days (range: 2–60). Patients with no symptoms (11 cases, 15%) had the shortest stay (median of 4 days) compared with patients presenting with three or more symptoms (9 cases, 13%), who remained in hospital longer (median of 22 days).

Admission for febrile neutropenia resulted in delay of subsequent administration of chemotherapy in 19 (26%) patients, complete cessation of chemotherapy in 5 (7%) patients and dose reduction in 10 (14%) patients. Of these, one was receiving adjuvant therapy and had already received primary prophylactic G-CSF. The remaining nine patients were being treated with palliative intent. The median age of all patients who had dose reductions was 68 years (range: 32–75).

In all, 4 (6%) patients died during their admission to hospital for febrile neutropenia. A 67 year-old lady with natural killer cell lymphoma died 25 days after admission. Her death was not attributed to febrile neutropenia but rather to co-morbidities, including congestive cardiac failure, pneumothorax and ultimately a perforated abdominal viscus. However, in the other three cases, neutropenic sepsis was deemed to be the main cause of death. These patients were: (1) a 59 year old with relapsed lymphoma admitted after his sixth cycle of palliative chemotherapy who died on the day after admission; (2) a 61 year old with acute myelogenous leukaemia receiving palliative chemotherapy who died 9 days after admission; and lastly, (3) a 59 year old patient with small cell lung cancer receiving second-line chemotherapy who died on the day of admission. In this last case, sepsis-related death occurred 12 days following commencement of cycle 1 of palliative second-line chemotherapy.

## Discussion

The NCAG recommendations set a benchmark for maintaining existing cancer services and for development and improvement where required. Neutropenic sepsis is one of the key areas highlighted and cancer networks must work to provide an acute oncology service with standardised protocols and policies, ensuring that patients receive antibiotics within one hour of presentation (door-to-needle; NCAG, 2009). Our study demonstrates that within SWLCN, chemotherapy-induced febrile neutropenia was generally recognised early and managed appropriately, however there remains room for improvement. Timely administration of appropriate antibiotics within 60 min requires particular attention. The seriousness of the condition is underlined by the fact that three patients died as a direct consequence of neutropenic sepsis, translating into a mortality rate of 4.2% for our series. This is in keeping with reported mortality rates of between 2 and 10% (Kuderer *et al*, 2006; Klastersky and Paesmans, 2007).

The NCEPOD report (NCEPOD, 2008) revealed similar problems in delayed administration of antibiotics, the use of incorrect antibiotics and delay in both diagnosis and senior staff review. Recommendations include access to a local policy and clinical care pathway, management by experienced staff, as well as close liaison with A and E departments. It was apparent in our study that there was no common protocol that existed between the hospitals of the network, and this may have contributed towards delayed management and use of antibiotics, but also accounts for the variation of diagnostics undertaken. The National Comprehensive Cancer Network (NCCN) guidelines (Aapro *et al*, 2006; Smith *et al*, 2006) recommend routine blood tests and blood cultures for all patients, but other investigations such as chest x-rays, dip-stick urinalysis, urine and stool cultures, are left at the discretion of the assessing clinician. As time is a highly significant factor in the successful management of neutropenic sepsis, a network-based protocol would aid in expediting assessment and early treatment.

Almost all patients included in our study had well-established risk factors for the development of febrile neutropenia. American and European guidelines (Aapro *et al*, 2006; Smith *et al*, 2006) acknowledge the following factors as significant for consideration of prophylactic administration of haematopoietic growth factors: (1) chemotherapy regimens with a risk of >20% for the development of febrile neutropenia or (2) regimens with a risk between 10 and 20% in conjunction with certain patient-related risk factors such as stage of disease and age. Of our patients that fulfilled either of these criteria, only 21% and 43%, respectively, received G-CSF (Table 5). Primary prophylactic G-CSF can reduce the risk of febrile neutropenia by 46% and infection-related mortality by 45%, whilst also allowing improved relative dose intensity of chemotherapy (Kuderer *et al*, 2007). In all, 50% of all episodes of febrile neutropenia occurred at or near the start of a chemotherapy course (cycles 1 and 2). This is in keeping with previous reports (Lyman, 2005), which led to the recommendation for starting prophylactic measures ‘upfront’ in appropriate risk groups (Aapro *et al*, 2006; Smith *et al*, 2006; NCCN, 2009). Identification of patients who will benefit from G-CSF and improving access to these agents is an important process before the commencement of chemotherapy.

Of note, one-third of patients with febrile neutropenia in our study were older than 65 years, and this may support the notion that increasing age is an independent predictor of the development of febrile neutropenia (Crawford *et al*, 2004; Aapro *et al*, 2006). There is sufficient evidence that prophylactic G-CSF reduces the incidence of chemotherapy-induced neutropenia, febrile neutropenia and infections in elderly patients (Repetto *et al*, 2003). Increased use of growth factors may be particularly relevant in the elderly, in order to optimise their cancer care and quality of life. Older patients are frequently under-treated because of misperceptions of their frailty; however dose reductions may compromise treatment efficacy (Pietropaolo *et al*, 2003; Lyman *et al*, 2004). Growth factors may help these management dilemmas.

Use of G-CSF in treatment of febrile neutropenia is more controversial and although 63% of our patients received G-CSF as secondary treatment, this may not necessarily be representative of other networks. The ASCO and EORTC guidelines recommend colony-stimulating factors as treatment only for patients who are at high risk for infection-associated complications. A meta-analysis (Clark *et al*, 2005) showed a significant reduction in the length of hospitalisation (hazard ratio: 0.63, 95% CI: 0.49–0.82, *P*=0.0006) with the use of G-CSF, but only a marginally significant decrease in infection-related mortality (odds ratio: 0.51, 95% CI: 0.26–1.00, *P*=0.05) and no significant reduction in overall mortality.

Primary antibiotic prophylaxis was used in 25% of cases in our study and its efficacy in reducing infections, febrile neutropenia and hospital admissions has been shown (Engels *et al*, 1998; Cullen *et al*, 2005). However there are valid concerns pertaining to possible increases in antimicrobial-resistant strains (Goossens *et al*, 2005), and widespread use is therefore discouraged for standard chemotherapy regimens in most solid tumours. Use may be considered for intermediate risk groups, such as lymphoma patients and treatments in which prolonged (7–10 days) neutropenia is anticipated (Cullen *et al*, 2007; NCCN, 2008).

It is important to recognise that prevention of neutropenic sepsis and its complications begins with appropriate, considered commencement of chemotherapy. Of particular concern from the NCEPOD was the finding that in 19% of cases, the decision to give chemotherapy was inappropriate, mostly on the grounds of progressive disease and abnormal blood results. Furthermore, in 27% of cases, chemotherapy was judged to have hastened or caused death, with the peak of deaths occurring 11–15 days after chemotherapy and likely related to the development of neutropenic sepsis (NCEPOD, 2008). For our data series, there was one case (1.4%) of death within 30 days of commencing chemotherapy, and with hindsight perhaps the use of second-line chemotherapy in this patient with metastatic SCLC was inappropriate. The NCAG recommendations provide a framework for a process of assessment, decision to treat, informed consent and prescription of chemotherapy, and emphasise the importance of detailed standardised consent forms and the involvement of senior trained oncology medical staff (NCAG, 2009). The evaluation of these aspects was beyond the scope of this study but will be an area of focus within the network.

Our study was also limited in being able to capture patients receiving chemotherapy at SWLCN hospitals but who presented with febrile neutropenia at hospitals outside the network. The NCEPOD reported that 15% of admissions were to hospitals other than the one where chemotherapy had been administered (NCEPOD, 2008). Peripheral hospitals may not be equipped with 24-h oncology services and similar levels of experience and it is therefore even more crucial for these sites to have access to well-designed protocols and to expert consultant advice. As outlined by NCAG, patient education regarding what to do in the event of a chemotherapy-induced complication is fundamental to ensuring they receive prompt appropriate care. Evaluating the efficacy of current patient education processes was also beyond the scope of this study, and in particular, it will be useful to assess the use of pre-designed ‘neutropenic sepsis’ cards which patients present to their local A and E in order to expedite appropriate management.

All patients in our study were treated with intravenous antibiotics and were admitted for inpatient care. Recent advances in the management of febrile neutropenia have highlighted the value of risk stratification and the evolving role of oral antibiotics with early hospital discharge in low-risk patients (Freifeld *et al*, 1999; Kern *et al*, 1999; Hughes *et al*, 2002). The Multinational Association for Supportive Care in Cancer has developed a risk index for the development of febrile neutropenia (Klastersky and Paesmans, 2007) and studies have shown that outpatient oral antibiotics are feasible in low-risk patients (Innes *et al*, 2003; Vidal *et al*, 2004; Klastersky and Paesmans, 2007). To our knowledge, this stratification is not routinely used in the UK (Innes *et al*, 2005) but will be an increasing area of interest, with important implications on decreased inpatient costs and improved patient convenience with outpatient care.

Undoubtedly there remain several aspects of cancer care that will require ongoing audit and review, and our own study highlights the areas that interact and affect the quality of care for one single chemotherapy-induced complication – febrile neutropenia. Our study has provided an important and detailed insight into the incidence and management of chemotherapy-induced febrile neutropenia in a representative cancer network in the United Kingdom. Neutropenic complications in cancer patients are associated with substantial morbidity, mortality and healthcare costs, warranting research and audit in this area.

We support NCAG's initiative to encourage chemotherapy services to undertake regular self-assessment procedures to identify gaps in their performance and take urgent steps to improve quality of care. Their recommendations in combination with the NCEPOD data have aided SWLCN in analysing the results of this study and putting them in perspective. Areas of potential improvement are currently being addressed for the hospitals within our cancer network, based on the results from this study and on the recommendations set out by NCAG (see Table 6). Improved uptake of prophylactic G-CSF according to ASCO and ESMO guidelines (particularly in patients above the age of 65), could reduce the risks of morbidity and mortality associated with neutropenic sepsis. Time intervals from arrival to completion of assessment and commencement of treatment for febrile neutropenia need improvement, and to achieve this, physician and nursing protocols to standardise and streamline clinical care pathways for the whole network are under consideration. It is hoped that the recommendation for NICE to provide a nationwide policy for management of neutropenic sepsis will lead to a standardised approach within and across networks. A dedicated specialist committee should review all clinical cases resulting in death following admission with febrile neutropenia. Finally, continued audit of current practices should be ongoing, in order to identify and rectify weaknesses whilst simultaneously consolidating current quality care.

## Figures and Tables

**Table 1:** Areas highlighted by NCEPOD in management of neutropenic sepsis

**Organisational**	**Clinical**	**Patient**
No neutropenic policy in A and E departments	Failure of junior doctors to make diagnosis	Insufficient information that risk of neutropenic sepsis may continue
Clinicians unaware of neutropenic sepsis policy	Lack of early assessment by senior medical staff	Patients not obtaining advice when unwell
Inappropriate place of care for neutropenic sepsis	Delayed admission to hospital and transfer to intensive care	
Infrequent visits to cancer units by oncologist	Unacceptable delay in resuscitation	
	Delayed antibiotic prescription and administration	
	Different antibiotics to those outlined in policy	
	Staff unaware neutropenic sepsis can occur without fever	

**Table 2:** Primary sites of underlying malignancies of cancer patients presenting with febrile neutropenia

**Underlying malignancy**	**Number of cases, *n*=71 (%)**
Breast cancer	13 (18)
Lymphoma	13 (18)
Other haematological malignancy	11 (15)
Sarcoma	9 (12)
Lung cancer	8 (11)
Lower gastrointestinal cancer	7 (10)
Other	6 (8)
Upper gastrointestinal cancer	4 (6)

**Table 3:** Presenting symptoms on admission

**Site of symptom**	**Number of cases, *n*=71 (%)**
Chest	35 (49)
Gastrointestinal	33 (46)
Mouth	13 (18)
Skin	9 (12)
Genitourinary	6 (8)
Neurological	3 (4)

**Table 4:** Prevalence of (level I/II evidence for) risk factors for the development of febrile neutropenia (according to ASCO and EORTC guidelines)

**Risk factor (level I/II evidence)**	**Number of cases, *n*=71 (%)**
Advanced disease	39 (54)
Age ⩾65 years	25 (35)
Previous neutropenic event	18 (25)
No prophylactic treatment (G-CSF and/or antibiotic treatment)	43 (60)
Chemotherapy ≥20% risk for febrile neutropenia	23 (32)

Abbreviations: ASCO=American Society of Clinical Oncology; EORTC=European Organisation of Research and Treatment of Cancer; G-CSF=granulocyte colony-stimulating factor.

**Table 5:** Risk stratification according to ASCO/EORTC guidelines and extent of prophylactic administration of G-CSF

	**Patients to be considered for G-CSF according to ASCO/EORTC guidelines**	**Number of eligible patients receiving G-CSF (%)**
Advanced disease	39	5 (13)
Regimen with 10–20% risk of febrile neutropenia and age ≥65	14	3 (21)
Regimen with 10–20% risk of febrile neutropenia and previous neutropenic event	14	5 (35)
Regimen >20% risk for febrile neutropenia	23	10 (43)

Abbreviations: ASCO=American Society of Clinical Oncology; EORTC=European Organisation of Research and Treatment of Cancer; G-CSF=granulocyte colony-stimulating factor.

**Table 6:** Key areas for improvement and action points for SWLCN

*Key areas for improvement*	
Improve time to antibiotics	
Develop a structured education and monitoring process for high risk patients	
Improve uptake of prophylactic antibiotics for high risk patients	

*Key actions*	
New clinical pathway – streamlined to avoid delays	
Within 10 min of presentation	Assessment and observations, blood tests and antibiotics sourced, senior designated person contacted
Within 1 h of presentation	Administration of intravenous antibiotic Medical assessment Decision on additional tests and ongoing management
Within 1–2 h of presentation	Ongoing monitoring and review
Within 4 h of presentation	Patients requiring inpatient stay are admitted to hospital
	
Development of acute oncology service within each cancer centre	
Development of standardised SWLCN protocol for management of neutropenic sepsis	
Patient information	
Reviewed and developed in partnership with patients; for example, the patient alert card to help patients understand when and how to access emergency help and advice	

Education and training package for staff	
Implementation of the HEAT (history, examine, action and treat tool; Dikken, 2009). This consists of a poster, patient alert card and DVD outlining symptoms of neutropenic sepsis and required actions	
Ongoing audit	

Abbreviations: DVD=digital video disc; SWLCN=South West London Cancer Network.
